# Natural Products-Based Metallic Nanoparticles as Antimicrobial Agents

**DOI:** 10.3389/fphar.2022.895616

**Published:** 2022-06-02

**Authors:** Deny Susanti, Muhammad Salahuddin Haris, Muhammad Taher, Junaidi Khotib

**Affiliations:** ^1^ Department of Chemistry, Kulliyyah of Science, International Islamic University Malaysia, Kuantan, Malaysia; ^2^ Department of Pharmaceutical Technology, Kulliyyah of Pharmacy, International Islamic University Malaysia, Kuantan, Malaysia; ^3^ IKOP Pharma Sdn Bhd, Jalan Sultan Ahmad Shah, Kuantan, Malaysia; ^4^ Pharmaceutics and Translational Research Group, Kulliyyah of Pharmacy, International Islamic University Malaysia, Kuantan, Malaysia; ^5^ Department of Pharmacy Practice, Faculty of Pharmacy, Airlangga University, Surabaya, Indonesia

**Keywords:** antimicrobial, nanoparticles, gold nanoparticle, silver nanoparticle, natural products, green synthesis

## Abstract

Natural products offer a wide range of bioactivity including antimicrobial properties. There are many reports showing the antimicrobial activities of phytochem icals from plants. However, the bioactivity is limited due to multidrug resistant properties of the microorganism and different composition of cell membrane. The antibacterial activity of the natural products is different toward Gram-negative and Gram-positive bacteria. These phenomena are caused by improper physicochemical conditions of the substance which hinder the phytochemical bioactivity against the broad range of bacteria. One of the strategies to improve the antimicrobial action is by biogenic synthesis *via* redox balance of the antimicrobial active substance with metal to form nanosized materials or nanoparticles (NPs). Antibiotic resistance is not relevant to NPs because the action of NPs is *via* direct contact with bacterial cell walls without the need of penetration into microbial cells. The NPs that have shown their effectiveness in preventing or overcoming biofilm formation such as silver-based nanoparticles (AgNPs), gold-based nanoparticles (AuNPs), platinum-based nanoparticles (PtNPs) and Zinc oxide-based nanoparticles (ZnONPs). Due to its considerably simple synthesis procedure has encouraged researchers to explore antimicrobial potency of metallic nanoparticles. Those metallic nanoparticles remarkably express synergistic effects against the microorganisms tested by affecting bacterial redox balance, thus disrupting their homeostasis. In this paper, we discuss the type of metallic nanoparticle which have been used to improve the antimicrobial activity of plant extract/constituents, preparation or synthesis process and characterisation of the plant-based metallic nanoparticles.

## Introduction

Antimicrobial resistance occurs when the pathogenic microorganisms survive upon exposure to a drug that would normally kill them and allows them to continue the infection (Oneil, 2016). The cases of antimicrobial resistance increase every year and estimate 10 million deaths every year (Oneil, 2016). Some bacteria are known to develop resistant such as methicillin-resistant *Staphylococcus aureus*, vancomycin resistant *Enterococci, Pseudomonas aeruginosa* and *Klebsiella pneumonia* as well as *Clostridium difficile* (Khan et al., 2015).

Antimicrobial resistance could be developed at DNA and protein levels (Wang et al., 2017). Antimicrobial resistance happens due to microbe’s interaction with their environment. Naturally, they will be able to develop intrinsic resistance to one or more antimicrobials. Whereas, in clinical setting, the resistance refers to acquired resistance in the microbial population that was previously susceptible to antimicrobial agents (Munita and Arias, 2016).

Antimicrobial from natural products have inspired antibiotic discovery and have been used for microbial control. These include plant extracts, small antimicrobial peptides, essential oils, bacteriocins and various groups of compounds (Stan et al., 2021). There are several reports showing the antimicrobial activity of free compounds isolated from natural sources. Natural products such as tannins are good substances to control microbial growth by interact with bacterial proteins and precipitating them (Puljula et al., 2020). Chalcone derivative from *Croton anisodontus* Mull. Arg. acts as a competitive inhibitor Mep A efflux pump and potentiates ciprofloxacin’s action against multidrug-resistant *Staphylococcus aureus* (Xavier 2020). Betulinic acid was reported to have string inhibition against *Candida albicans* (Lashin et al., 2021). Nimbolide from *Azadiracta indica* A. Juss possesses significant bactericidal activity against *Helicobacter pylori* by killing free living bacteria and cells within biofilm (Wylie et al., 2022). The following table (Table 1) presents selected antimicrobial plants which were obtained from database using the keywords of “plant” and “antimicrobial activity”.

**TABLE 1:** Plants with antimicrobial activity.

Plant	Family	Compounds	MIC	Action	Microorganism	Ref
*Morus mesozygia* Stapf ex A.Chev	Moraceae	3β-acetoxyurs-12-en-11-one moracin Q, moracin T, morasin R morasin U, moracin C, moracin M, artocarpesin, cycloartocarpesin	5–625 μg/ml	Not reported	*Citrobacter freund, Escherichia coli, Shigella dysenteri, Pseudomonas aeruginosa, Klebsiella pneumoniae, Salmonella typhii, Bacillus cereus, Staphylococcus aureus Streptococcus faecalis, Candida albicans, Microsporum audouinii*	Kuete et al. (2009)
*Citrus x sinensis* (L.) Osbeck	Rutaceae	pectin	0.162–3.125 mg/ml	Still not understood	*Shigella vulgaris, S. typhi, S. paratyphi, S. typhimurium, K. aerogenes, E. coli, Proteus vulgaris, Bordetella bronchiseptica, Vibrio cholerae, P. aeruginosa*	Ciriminna et al. (2020)
*Hypericum roeperianum* G.W. Schimp. ex A.Rich. var. Roeperianum	Hypericaceae	acetone extract	0.06–0.32 mg/ml	Not reported	*E. coli, S. typhimurium, P. aeruginosa, S. aureus, Enterococcus faecalis, B. cereus*	Elisha et al. (2017)
*Cremaspora triflora* (Thonn.) K.Schum	Rubiaceae	acetone extract	0.02–0.32 mg/ml	Not reported	*E. coli, S. typhimurium, P. aeruginosa, S. aureus, E. faecalis, Bacillus cereus*	Elisha et al. (2017)
*Maesa lanceolata* Forssk	Primulaceae	acetone extract	0.04–0.16 mg/ml	Not reported	*E. coli, S. typhimurium, P. aeruginosa, S. aureus, E. faecalis, B. cereus*	Elisha et al. (2017)
*Scutellaria baicalensis* Georgi	Lamiaceae	baicalein and wogonin	0.03–0.23 mM	Plasmatic membrane disintegration DNA fragmentation Accumulation of ROS Changes at the ultrastructural level	*Trichophyton rubrum, Trichophyton mentagrophytes, Aspergillus fumigatus, C. albicans*	Da et al. (2019)
*Cymbopogon citratus* (DC.) Stapf	Poaceae	citronellal	1.2 mg/ml	Disrupts cell membrane homeostasis Oxidative and genotoxic effects via ROS formation Inhibits biofilm formation	*C. albicans, C. glabrata*, *C. tropicalis*, *C. parapsilosis*, *C. krusei*	Singh et al. (2016)
Essential oil (Different plant extracts)	—	carvacol, thymol, eugenol, methyl eugenol	125–1250 μg/ml	Anti-adherence activity Anti-proteinase activity	*C. albicans, C. auris*	Shaban et al. (2020)
*Cinnamomum verum* J. Presl	Lauraceae	cinnamaldehyde	1000 μg/ml	Inhibit biofilm formation	*Streptococcus mutan*	He et al. (2019)
Berberis cretica L	Berberidaceae	magnoflorine	50–100 μg/ml	Inhibit biofilm formation	*C. albicans C. tropicalis* var. *tropicalis C. parapsilosis* var. *parapsilosis, C. glabrata*	Kim et al. (2018)
						Okon et al. (2020)
*Eucalyptus robusta* Smith	Myrtaceae	(+)-eucalrobusone X	10.78 μg/ml	Not reported	*C. albicans*	Shang et al. (2019)
*E. robusta* Smith	Myrtaceae	eucalrobusone U	1.53 μg/ml	Not reported	*C. glabrata*	Shang et al. (2019)
*Laurus nobilis* L	Lauraceae	essential oil	250–500 μg/ml	Inhibits cell wall formation Affects membrane ionic permeability	*C. albicans,C. tropicalis, C. krusei*	Peixoto et al. (2017)
*Punica granatum* L	Lythraceae	gallic acid	6.25–100 μg/ml	Inhibition of ergosterol biosynthesis Reduction of squalene epoxidase activity	*Trichophyton rubrum T. mentagrophytes, T. violaceum, Microsporum canis, T. verrucosum, T. schoenleinii; C. glabrata, C. albicans, C. tropicalis*	Li et al. (2017)
*Polygonum cuspidatum* Sieb. et Zucc	Polygonaceae	resveratrol	20 μM	Fungal cell apoptosis via caspase-dependent pathway	*C. albicans*	Wang et al. (2013)
						Lee and Lee, (2015)

### Role of NPs in Biofilm Formation

NPs has been recorded as an effective method in combating microbial resistance and multidrug-resistance mutant (Wang et al., 2017) especially on the interaction with microbial biofilm.

Bacterial biofilms are complex surface on the communities of bacteria that consist of microorganism culture, extracellular polymer matrix (EPM), complex biopolysaccharide, secreted protein proteins and extracellular DNA (Muhammad et al., 2020; Shkodenko et al., 2020).

**FIGURE 1:**
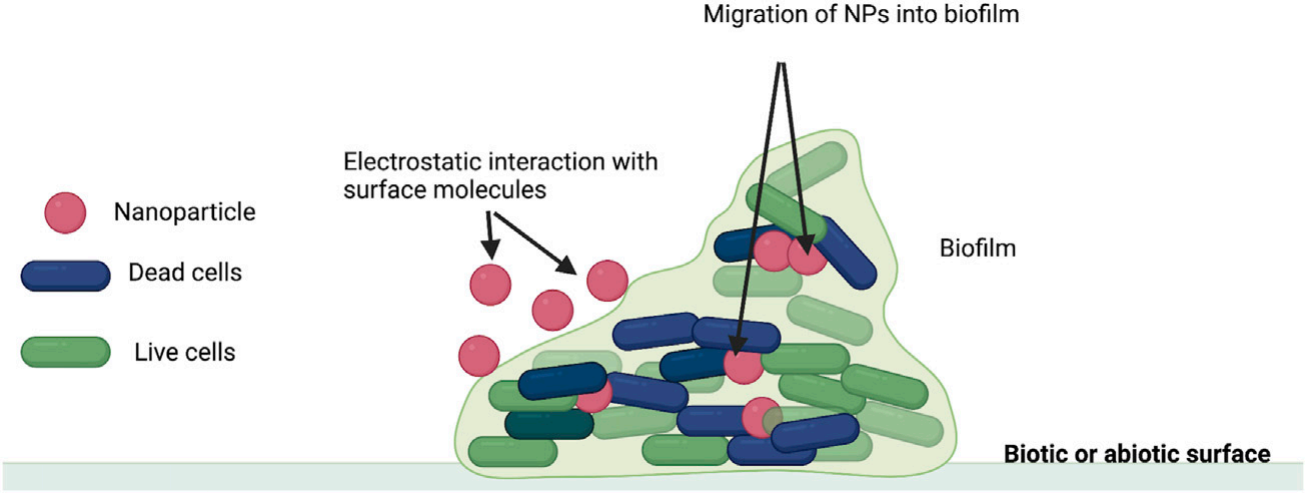
Interaction between biofilm and NPs (Adapted from Shkodenko et al., 2020).

The role of NPs in antimicrobial activity including the disruption of bacterial membranes and interaction with biofilm (Shkodenko et al., 2020). The interaction between NPs and biofilm could occur in following step: transfer of NPs to the biofilm; attachment to the surface and migration into biofilms (Figure 1). The process is affected by physicochemical properties of NPs, the environment and extracellular polymeric matric (Shkodenko et al., 2020).

### Nanoparticle in Drug Delivery

New approach to combat resistant microorganisms is urgently required to reduce the clinical burden in the use of antibiotics. Nanotechnology-based antimicrobials could be one of strategies which is acting synergistically *via* intrinsically antimicrobials and those as carriers (Makvandi et al., 2020). Nanotechnology is emerging field of science which deals with the synthesis of nanoparticles in improving physicochemical properties of materials for human benefits (Chau et al., 2022). The success research in nanoparticle-based delivery is due to their advantages such as safety assessment, scalable production, and availability for clinical trials (Hagbani et al., 2022).

The nanoparticles fall between 1 and 100 nm size which can be prepared by top-down methods include mechanical milling, laser ablation, etching, sputtering, and electro-explosion and bottom-up approaches such as chemical vapor deposition, solvothermal and hydrothermal methods, sol–gel method, soft and hard templating methods, reverse micelle methods (Baig et al., 2021). Nanoscale delivery has gained interest due to their exceptional activity allowing reaction to be achieved effectively and efficiently (Baig et al., 2021). Metal-based nanomaterials have been continuously investigated for their application due to their potential usage. Metal nanoparticles (MNPs) display potential capacity and higher surface area making them suitable in numerous applications including medicine, drug delivery, cosmetics, food product, sensors, optics, electronics, paints and agriculture (Perveen et al., 2022).

The metal-based nanocarriers have been reported to increase the antimicrobial activity (Makvandi et al., 2020) in addition to their own activity (Yonathan et al., 2022). The use of noble (magnetic) metal nanoparticles (MNPS) especially gold (Au), silver (Ag) and platinum (Pt) have been attracting great attention due to its multiple antibacterial properties and simple synthesis methods (Shafey, 2020). They act selectively attached to a functional molecule and allow transportation to targeted locations under magnetic fields (Ahmad et al., 2022). The noble metal particle can kill bacteria and eradicate biofilms by cell membrane potential stability disruption as well as binding bacterial enzymes or DNA and (Ye et al., 2022). MNPs can also act as free radical damaging bacterial cell membrane (Ye et al., 2022). Meanwhile, the antibacterial agents show similar mechanisms with one or multiple targets on bacterial protein synthesis, bacterial cell wall synthesis, bacterial cell membrane destruction, bacterial DNA replication and repair as well as inhibition of metabolite pathway (Khameneh et al., 2019). When the antimicrobial agent is encapsulated with metal-nanoparticles, they would have a synergistic effect therefore the toxicity level would decrease and the efficacy would increase.

### MNPs in Antibiotic Potency Enhancement

Metal nanoparticles are becoming a trend strategy to improve antibiotic activity due to its simple synthesis. Antibacterial activity of the antibiotic is different against bacterial gram stain, due to organization of the structures outside the plasma membrane but below the capsule where in Gram-negative organisms these structures constitute the cell envelope, whereas in Gram-positive organisms they are called a cell wall (Salton and Kim, 1996). The silver nanoparticles alone excellently discompose the polymer sub-units of the cell membrane in micro-organisms. In combination with plant material which is mediated silver nanoparticles consequently rupture the cell membrane and destroy the protein synthesis mechanism in the bacteria (Bukhari et al., 2021).

Study on antibiotic colistin on anionic gold nanoparticle against *Escherichia coli* reduces minimum inhibition of 6-folds compared to colistin alone (Fuller et al., 2020). A gold nanoparticle of vancomycin has shown more effective inhibition towards Gram-negative bacteria of *E. coli*, *Klebsiella oxytoca* and *Pseudomonas aeruginosa* with 1.4, 1.6 and 1.8 folds inhibition and towards Gram-positive bacteria, *Staphylococcus aureus* with 1.6 fold compared to free vancomycin (Hagbani et al., 2022). Combinatorial silver nanopacked Daptomycin inherits synergistic effects of both for their bactericidal activity (Zheng et al., 2016).

### Plant Extract Encapsulated With MNPs

#### Gold Nanoparticle in Antimicrobial Delivery of Natural Products

Gold nanoparticles (AuNPs) is an important class of nanoparticles. They have been widely used in medical and non-medical applications as an ideal material for a variety of purposes. Inertness, biocompatibility, and, most importantly, low toxicity. Au-NPs have been shown to have biological activity and have been studied using a variety of important Au (III) properties that aid in Au-NPs synthesised from a variety of materials. Au-NPs are adaptive, resourceful, and can be designed to execute a wide range of tasks, making them a viable alternative to Au. In comparison to other NPs such as Ag, Au-NPs are more effective at attacking a variety of bacteria and viruses (Aljarba et al., 2022).

While Au is widely used as a nontoxic nanomaterial, the materials used to prepare and modify Au-NPs may be toxic (Guliani et al., 2021). While the presence of Au-NPs in high concentrations may indicate toxicity, there are many studies showing that Au-NPs do appear to possess antibacterial properties. At certain concentrations, it has been demonstrated that these NPs are non-toxic to normal cells (Guliani et al., 2021). Not only do modified Au-NPs have great antimicrobial abilities against standard strains of bacteria, but they also have a unique ability to fight cancer (Aljarba et al., 2022). Au (III) that is attached to different drug systems has been shown to make them more effective at killing bacteria. A lot of different bacteria, like *M. luteus, E. coli, S. aureus,* and *P. aeruginosa* can be killed by aminoglycosides that are coated with AuNPs (Bera and Mondal, 2022).

Antimicrobial agents are used to keep moulds, bacteria, and fungi populations under control. To prevent infection, these substances prevent bacteria from multiplying on various surfaces. Au and Ag are two examples of antimicrobial agents that contain active metal ingredients. Numerous studies have established the antimicrobial effect of gold nanoparticles (Hammami et al., 2021). Due to the fact that NPs’ antimicrobial activity is inversely proportional to their size, the smaller the size of Au-NPs, the higher their antimicrobial activity against a variety of microbes such as *K. pneumonia, S. aureus, P. vulgaris, E. coli,* and *B. subtilis.* Thangamani and Bhuvaneshwari investigated these synthesised Au-NPs’ antimicrobial activity against the aforementioned microbes (Thangamani and Bhuvaneshwari, 2019). In addition, antibacterial activity of NPs has been investigated against a variety of microbes, including *E. coli, E. faecalis, S. typhimurium, P. aeruginosa, Vibrio damsel, Vibrio fluvialis* and *Candida albicans* (Sunderam et al., 2019). The biological activity of Au-NPs synthesised *via* biogenic synthesis using the endophytic fungus *Cladosporium cladosporioides* derived from seaweed *Sargassum wightii* was evaluated against *S. aureus, E. coli, P. aeruginosa* and *B. subtilis*. Among the reported microbes, these AuNPs portrayed the lowest activity towards *B. subtilis* and the greatest effect toward *S. aureus*. After 3 min, approximately half of the *E. coli* were died, while 80% were killed after 6 min. After 10 minutes of photoactivation, no bacterial stains were detected (Manjunath et al., 2017).

Formulating NPs from natural extracts containing biologically active pharmacophores is common. Rajathi and colleagues synthesised Au-NPs containing *Stoechospermum marginatum* and evaluated the antimicrobial activity against *P. aeruginosa, Klebsiella oxytoca, Salmonella typhimurium, Enterobacter faecalis, Klebsiella pneumonia* and *Proteus vulgaris*. They discovered that *Klebsiella pneumonia* was more susceptible to these AuNPs, among other microbes (Rajathi et al., 2012). Dual metallic nanoparticles were synthesised by combining Ag and Au in various ratios using a red alga called *Gracilaria sp*. These NPs were then tested for antimicrobial activity against a variety of bacteria, including *Salmonella typhii, E. coli, S. aureus* and *K. pneumonia*. These NPs have bactericidal effects on *S. aureus* and *K. pneumonia* (Ramakritinan et al., 2013). Additionally, Au-NPs from *Morus alba* L. (mulberry) leaf extract were also synthesised and tested against human pathogens. These Au-NPs successfully inhibited *Vibrio cholera* and *S. aureus* (Adavallan and Krishnakumar, 2014). Au-NPs were synthesised from HAuCl_4_ using a papaya leaf extract *via* a green synthetic technique. Investigating these NPs for biomedical applications revealed that they are effective against *S. aureus* and *P. putida* (Sunkari et al., 2017).

The Au-NPs from *Elaeocarpus ganitrus* Blume seed extract via a hydrothermal route were synthesised and their antimicrobial activity was observed against *Proteus desmolyticum* and *S. aureus*, in which both of the microbes were killed (Vinay et al., 2021). Another group of researchers synthesised Au-NPs from *Mentha longifolia* (L.) L. extract and demonstrated their efficacy in killing *B. subtilis*, *K. pneumoniae* and *S. aureus* strains (Rauf et al., 2021). Biological Au-NPs containing *Jasminum auriculatum* Vahl. leaf extract exhibited promising anti-bacterial activity against *E. coli*, *S. aureus, Streptococcus pyogenes*, and *K. pneumonia*) (Balasubramanian et al., 2020). Additionally, Au-NPs were synthesised from a mixture of *Catharanthus roseus* (L.) G. Don and *Carica papaya* L. leaf extracts and these NPs possessed antibacterial activity against *E. coli*, *Bacillus subtilis*, *S. aureus* and *Proteus vulgaris* (Muthukumar et al., 2016).

Antibiotic resistance has emerged as a major global issue, thus reducing the availability of effective antibiotics. As a result, novel antibiotics from various sources especially natural extracts with novel delivery are urgently required (Wang et al., 2017) The Au-NPs produced using H_2_O_2_ as a reduction enhancer and starch as a reducing agent exhibited significant bactericidal activity against *S. aureus* (Emam et al., 2017). Functionalised Au-NPs with arginine and hydroxyapatite portrayed antibacterial activity against three bacteria, *S. aureus, P. aeruginosa* and *E. coli.* Besides, it was discovered that the activity of these functionalized Au-NPs was similar to that of Au-NPs, but with a stronger inhibitory effect towards *P. aeruginosa* strain (Kurtjak et al., 2017).

*P. aeruginosa* is believed to be the most resistant bacteria due to its mutational capability, demonstrating a high degree of resistance to antibiotics. The Au-NPs were formulated with curcumin extracted from *Curcuma pseudomontana* J. Graham and hematite (a-Fe_2_O_3_). It was reported that these NPs showed antimicrobial action against *P. aeruginosa, S. aureus* and *B. subtilis* with a higher degree of antimicrobial effect toward *E. coli* (Muniyappan et al., 2021). Lists of the Au-NPs containing natural extracts and demonstrates antibacterial activity is presented in Table 2.

**TABLE 2:** Lists the Au-NPs containing natural extracts and demonstrates antibacterial activity.

Nanoparticle	Plant	Extract/Compounds	Microorganisms	Gram Strain	Size (nm)	Activity	Ref
Au	*Curcuma pseudomontana* J. Graham	Extract (Rhizomes)	*S. aureus, B. subtilis, P. aeruginosa* and *E. coli*	Positive and negative	20 to 39	At 300 microg/ml concentration, zone nhibition	Muniyappan et al. (2021)
						*E. coli* (28 mm)	
						*B. subtilis* (26 mm)	
						*S. aureus* (25 mm)	
						*P. aeruginosa* (23 mm)	
Au	*Annacardium occidentale* L	Extract (Leaves)	*B. subtilis* and *E. coli*	Positive and negative	10 to 30	At 40 microL, zone inhibition: *E. coli* (24 mm)	Sunderam et al. (2019)
						*B. subtilis* (10 mm)	
Au	*Dracocephalum kotschyi* Boiss	Extract (Leaves)	S*. aureus, B. subtilis, B. cereus, E. coli* and *P. aeruginosa*	Positive and negative	7.9 to 22.63	No antibacterial activity	Dorosti and Jamshidi, (2016)
Au	*Jasminum auriculatum* Vahl	Extract (Leaves)	*S. pyogenes, S. aureus, E. coli* and *K. pneumonia*	Positive and negative	8 to 37	At 30 microL, inhibition zone: *S. pyogenes* (12 mm) *S. aureus* (9 mm)	(Balasubramanian et al., 2020))
						*E. coli* (12 mm)	
						*K. pneumonia* (7 mm)	
Au	*Pimpinella anisum* L	Extract (Seeds)	*S. aureus* and *E.coli*	Positive and negative	63	No antibacterial activity	Zayed et al. (2020)
Au	*Glycyrrhzia glabra* L	Extract (Roots)	*B. subtitis, S. aureus, P. aeruginosa* and *Salmonella typhi*	Positive and negative	53.7	Good antibacterial activity against gram negative microbes	Al-Radadi, (2021)
Au	*Pituranthos tortuosus* (Desh.) Benth. and Hook. F. ex. Asch. and Schweif	Extract (Aerial)	*Helicobacter pylori*	Negative	5 to 15	AuNPs showed antibacterial activity towards *H. pylori* with MIC value of 15, 63 (microgram/ml)	Abd El-Moaty et al. (2021)
Au	*Pongammia pinnata* (L.) Pierre	Extract (Leaves)	*Mycobacterium tuberculosis*	Acid-fast gram positive	16	Effective in killing	Govindaraju et al. (2020)
						*M. tuberculosis* with MIC of 10 microg/ml	
Au	*Stoechospermum marginatum (kutzing)*	*Extract (Leaves)*	*P. aeruginosa, K. oxytoca, E. faecalis, K. pneumonia, E. coli, S. typhi, S paratyphi*	Positive and negative	18.7–93.7	Excellent activity in comparison to positive control (tetracyclines) towards *E. faecalis*	Rajathi et al. (2012)
Au	*Glaciracia sp.*	Extract (Red alga)	*S. aureus, E.coli, K. pneumonia, S. typhi, P. aeruginosa*	Positive and negative	20–40	Bimetallic NP’s of 1:3 concentration showed zones of inhibition against the pathogenic bacteria such as *Staphylococcus aureus and Klebsiella pneumoniae* rather than Ag NPs and Au NP’s	Ramakritinan et al. (2013)
Au	*Mentha longifolia* (L.) L	Extact (Leaves)	*K. pneumonia, S. aureus, B. subtilis*	Positive and negative	13.45	AuNPs possessed antibacterial activity towards *S. aureus*	Rauf et al. (2021)
Au	*Moringa oleifera* Lam	Extract (Bark, leaf and flower)	*Aspergillus sp.*	—	-(non-uniform spherical)	Effective in killing	Mondal et al. (2022)
						*Aspergillus sp*. at high concentration (200 mg/L)	

#### Silver and Other Nanoparticles in Antimicrobial Delivery of Natural Products

Silver nanoparticles (AgNPs) showed remarkable biological and physicochemical properties. Silver ions demonstrated antimicrobial activity. The use of AgNPs in encapsulating natural products material will enhance their antimicrobial activity *via* DNA activity (Ahmad et al., 2022).

*Euphorbia serpens* Kunth synthetized using green synthesis AgNPs showed significant antibacterial effect against against *E. coli* with 20 ± 06 mm and *S. typhi* with 18 ± 0:5 mm zone of inhibition compared to standard antibiotic amoxicillin with 23 ± 0:3 mm and 20 ± 0:4 mm zone of inhibition (Ahmad et al., 2022). *Trigonella foenum-graecum*-AgNPs was reported to have antimicrobial activity against pathogenic fungi in plant *Alternaria alternata* and pathogenic bacteria in plant *Pseudomonas syringae* (Khan et al., 2019).

Biogenic synthesis of *Persicaria odorata* (Lour.) Sojak silver nanoparticles corroborated improve the bactericidal activity by dose-dependent inhibition against *S. epidermis* and Methicillin resistant *S. aureus* (Lubis et al., 2022). A comparison study of antibacterial activity between AgNPS and Ag zeolite A (ZA) showed that AgNPS against *E. coli* ATCC 11229 and *S. aureus* ATCC found that the inhibition of AgNPs is higher than AgZA (Asraf et al., 2022). AgNPs of *Mentha piperita* L. increased the antibacterial activity of the extracts in inhibiting the growth of both *S. aureus* and *P. aeruginosa* (Mojally et al., 2022). The synergistic effect of ZnO nanoparticle with plant extract was shown by *Agle marmelos* (L.) Correa (Am-ZnO) which shown greater antibacterial and antibiofilm effects against Gram-negative bacteria when compared to Gram-positive bacteria (Senthamarai and Malaikozhundan, 2022). Interestingly the *Sambucus ebulus* L.-AgNPs able to inhibit Gram-negative bacteria as reported by *S. aureus* with MIC value of 1.5 mg/ml. (Hashemi et al., 2022a). Table 3 displays the silver and other metal nanoparticles which were recently reported.

**TABLE 3:** The synthesis of metallic nanoparticle in plant-based antimicrobial.

Nanoparticle	Plant	Extract/Compounds	Microorganisms	Gram Strain	Size	Activity	Ref
Ag	*Ferula ovina* Boiss	Extract	*E. coli* and *S. typhimurium*	Negative	15.7–23.86 nm	Enhanced	Allafchian et al. (2022)
Ag	*Glochidion candolleanum* (Wight and Arn.) Chakrab. and M. Gangop	ethyl acetate extract	*Salmonella enterica*	Negative	N/A	Enhanced	Balachandar et al. (2022)
			*P.aeruginosa*				
Ag	*Berberis vulgaris* L	Aqueous extract	*P. mirabilis, E. coli, E. faecalis S. aureus, A. baumannii, P. aeruginosa and K. pneumonia*	Negative and positive	45–60 nm	Enhanced	Hashemi et al. (2022b)
Ag	*Sambucus ebulus* L	Methanol	*S. aureus*	Positive	35–50 nm	Enhanced	Hashemi et al. (2022a)
Ag	*Areca catechu* L	Aqueous		Positive and negative	15–20 nm	Enhanced	Hu et al. (2022)
Ag	*Pisum sativum L*	Aqueous	*S. aureus* ATCC 25923 (22 ± 2 mm)	Positive	30 nm	Enhanced	Alarjani et al. (2022)
Ag	*Passiflora subpeltata* Ortega	Aqueous	*B. cereus* (27.5 mm)	Positive	22.6 nm	Enhanced	Loganathan et al. (2022)
Ag	*Guettarda apeciosa* L	Aqueous leaf extract	[Staphylococcus Aures (MTCC 25,923), Basillus Subtilis (MTCC 2451)) and two gram-negative (Escherichia coli (MTCC 25,922), Streptococcus Aureus (MTCC 273)]	Positive and negative	6.5–160 nm	Antimicrobial activity	Deivanathan and Prakash, (2022)
Ag	*Anagallis monellin* L	Ethyl acetate leaves extract	*Staphylococcus aureus* and *Micrococcus luteus* with 15 and 16 mm	Positive and fungi	22 nm	Enhanced	Dridi et al. (2022)
			*Candida albicans*				
Ag	*Tricholoma ustale* Benth. and *Agaricus arvensis* L	Extract	*Pseudomonas aeruginosa, Klebsiella pneumonia, Staphylococcus aureus, Enterococcus faecalis, Candida albicans*, and *Candida utilis*	Negative, positive and fungi	20 nm	Antimicrobial activity	Kaplan et al. (2022)
Zn	*Aegle marmelos* (L.) Correa	Aquouse unripe fruit extract	Gram-negative bacteria	Negative	22.5 nm	Synergistic effect	Senthamarai and Malaikozhundan, (2022)
Ti	*Luffa acutangular* (L.) Roxb	Aqueous	*E. coli* (45 ± 0.21), followed by *P. aeruginosa* (43 ± 0.45), *S. aureus* (42 ± 0.13), *K. pneumoniae* (27 ± 0.54), *E. faecalis* (21 ± 0.41) and *B. subtilis* (18 ± 0.56) at 40 mg/ml concentration	Negative and positive	10–59 nm	Enhanced	Anbumani et al. (2022)
Fe	*Plumeria obtusa* L	Water Extract	*E. coli*, *B. subtilis* and fungal strains *A. niger* and *S. commune*	Negative and fungi	50 nm	Enhanced	Perveen et al. (2022)

### Green Synthesis MNPs

Several methods have been introduced to synthesise metal NPs including chemical, physical and biological methods using microwave-assisted, radiation-assisted, thermal decomposition, chemical and photo-chemical reaction, and green synthesis methods. Synthesis using bio-organisms or green synthesis compatible with the green chemistry principle. And green synthesis is required to avoid the production of unwanted or harmful by-products through the build-up of reliable, sustainable, and eco-friendly synthesis procedures. To achieve the goal of green synthesis, the natural resources and the ideal solvent systems that are environmentally friendly, non-toxic and safe reagent are essential (Singh et al., 2018).

Green synthesis of MNPs synthesised using biological techniques or green technology have diverse natures, with greater stability and appropriate dimension since they are synthesised using a one-step procedure. Therefore, the green synthesis of MNPs has been adopted to accommodate various biological materials, such as viruses, bacteria, fungi, algae, yeast, plant and plant products. Many researchers prefer to use green synthesis due to some advantages including cost effectiveness because the biological component itself acts as reducing and capping agents, eco-friendly approach as toxic chemicals are not used, and external experimental conditions like high energy and high pressure are not required that lead to energy saving process. Furthermore, the synthesis of a wide range of MNPs has been reported using various plants because it is a simple and easy process to produce MNPs at large scale compared to bacteria and/or fungi mediated synthesis (Song and Kim, 2009; Alarjani et al., 2022; Ye et al., 2022).

Plant mediated synthesis of MNPs is a revolutionary technique that has a wide range of applications in medicine, agriculture and food industries that offer an advantage which increases the life span of NPs. This is due to the physico-chemical properties of the plants based MNPs that overcome the limitation of conventional chemical and physical methods of NPs synthesis (Malik et al., 2014; Kalpana and Devi Rajeswari, 2018). Plant mediated synthesis of MNPs can be achieved by three different methods, intracellularly (inside the plant), extracellularly (using plant extracts), and using individual phytochemicals. Plants can mediate the synthesis of MNPs intracellularly because plants have the capability to accumulate the metals and transform these accumulated metals to NPs intracellularly. Moreover, the presence of several biomolecules such as protein, amino acids, carbohydrates, reducing sugar, vitamins, alkaloids, aldehydes, flavones, ketones, phenolics, saponins, tannins, and terpenoids in the plant plays a key role in the reduction of metals. These biomolecules contain hydroxyl, carbonyl and amine functional groups that react with metal ions and reduce their size into nano size. For example, flavonoids contain several functional groups, and it is believed that the -OH group of flavonoids is responsible for the reduction of metal ions into NPs (Marslin et al., 2018; Dikshit et al., 2021). Flavonoids not only help in bio-reduction of the metal ion to the nano size, but also play an important role in the capping of the NPs which is important for the stability and biocompatibility (Javed et al., 2020). In addition, reducing agents such as phenolics, sterols and alkaloids can reduce the metal ions into NPs in a single reaction (Biswas et al., 2013).

The type and nature of the metal used for MNPs biosynthesis mainly determine the NPs end use industry. Several metal such as silver (Ag), copper (Cu), gold (Au) and many others have been widely used for the biosynthesis of NPs using plant extracts of various plant species (Zhang et al., 2020). Au and Ag were the first metal ion used in plant extract-mediated synthesis of NPs. *Acalypha indica*, *Aloe barbadensis*, *Datura metel*, *Nelumbo nucifera*, *Jatropha curcas*, *Ocimum* leaf, *Cassia auriculata*, and *Rhododedendron dauricam* have been used to produce Ag nanoparticles. Gold nanoparticles have also been synthesized using *Aloe barbadensis* Miller, *Medicago sativa*, *Magnolia kobus*, *Diopyros kaki*, *Cinnamomum camphora*, and *Pelargonium graveolens* leaf (Jeevanandam et al., 2016).

Peralta-Videa et al., 2016 reported the synthesis of plant based metallic nanoparticles and their potential application. Metals from their constituents are reduced and stabilised by macromolecules and phytonutrients, such as proteins, reducing sugars, phenolics, flavonoids, ethyl alcohols, terpenes, and phenolic acids present in plant extracts. (Naseer et al., 2020) split the function of biomolecules in the formation of NPs as redoxed intermediaries for metals reductions and capped agents for non-agglomeration and post-surface modification of NPs.

In general, the synthesis of MNPs is initiated by mixing the plant biomass/extract with of noble metal salt precursors with biomaterial at a desired temperature and pH. The presence of various compounds of biomolecules act as reducing and capping agents for the synthesis of NPs from its metal salt precursors. The reduction of metal salt precursors to its successive NPs can be initially confirmed by visualising the colour change of the colloidal solution (Kuppusamy et al., 2016; Sriramulu et al., 2020).

**FIGURE 2:**
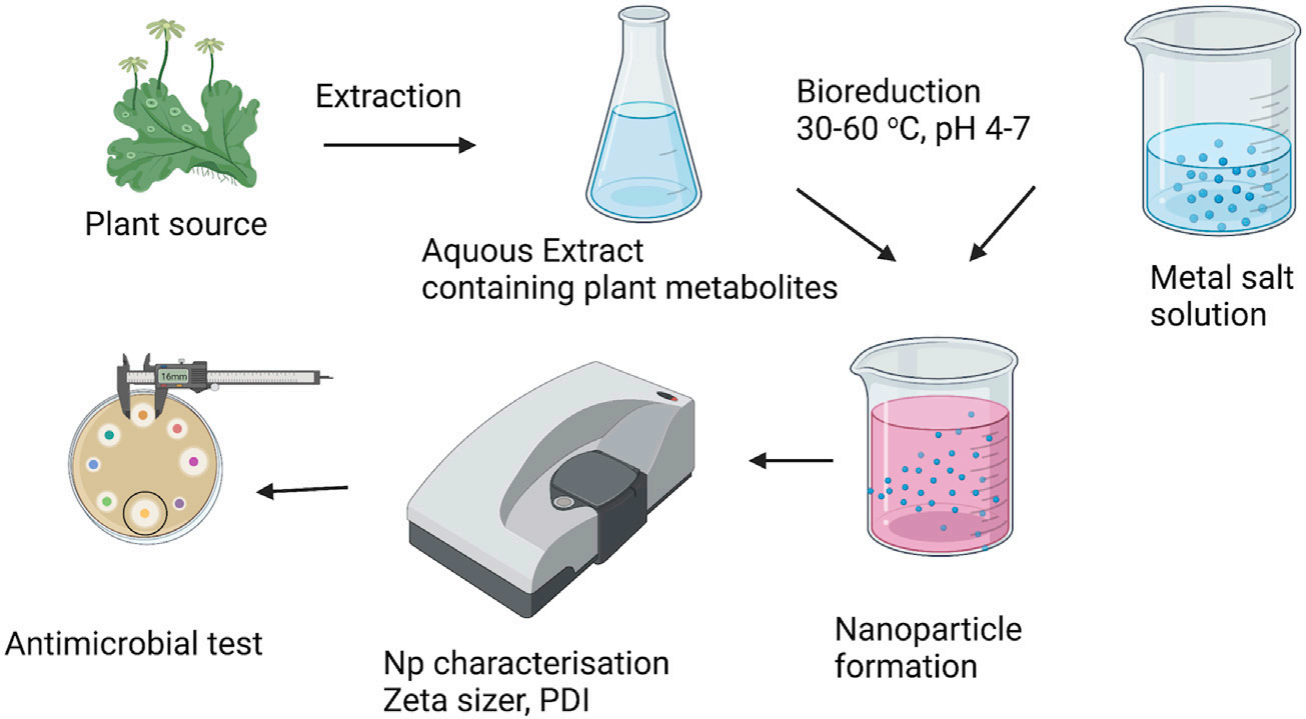
Schematic representation for plant-mediated biosynthesis of nanoparticles.

The variation in the size, shape, and properties of accumulated NPs can be observed due to the variation in stabilising and reducing potential of biomolecules present in the plant. For example, the formation of gold NPs inside the living plant, alfalfa was reported by (Gardea-Torresdey et al., 2002) when the plants were grown in AuCl_4_ rich environment. And in a similar kind of study, (Bali and Harris, 2010) observed the ability of *Medicago sativa* and *Brassica juncea* plants to accumulate Au NPs from aqueous solutions of KAuCl_4_. The NPs were majorly located in the xylem parenchyma cells while some were also accumulated throughout the epidermis, cortex, and vascular tissue. Several studies reported the synthesis of Ag, Au, Cu, Pt, Cd, Pt, Pd, Ru, Rh, etc. using various biological agents in the recent past. The experimental procedure for the synthesis of NPs using plant biomass is depicted in Figure 2.

### Unique Antibacterial Mechanism of MNPs

The mechanism of action of MNPs on bacterial cells is affected by the physiochemical properties of NPs such as charge, size, zeta potential, surface morphology and crystal structure. Whereas, the environmental factor, exposure time and bacterial strains are also influence the antibacterial effect of MNPs (Wang et al., 2017). There are three well known antimicrobial effects of NPs includes electrostatic interaction causing mechanical damage of cell walls, generation of reactive oxygen species resulting oxidative stress and disruption of cell structures and protein structure as a result of metal release (Wang et al., 2017).

Despite several approaches that have been made over the years, the precise mechanism of action of MNPs as antimicrobial agents is still not fully understood. Antimicrobial action of MNPs is linked to four main mechanisms, attraction to bacterial surface, destabilisation of bacterial or fungal cell wall and/or membrane with change in its permeability, induction of toxicity and oxidative stress by generation of reactive oxygen species (ROS) and free radical, and modulation of signal transduction pathways (Dakal et al., 2016). The adhesion of MNPs onto the surface of bacteria is the first step of a complex mechanism of bacterial inhibition. The adhesion of MNPs is influenced highly by their size and also zeta potential that depends on the method for their synthesis that might have a positive, negative or neutral surface charge. Varying surface charge of MNPs will cause the fluctuation of the antibacterial activity. Since the bacterial surface shows a slightly negative charge, positively charged MNPs are strongly attracted to the surface of bacteria, resulting in an increasing of antibacterial activity. On the other hand, negative or neutral charged MNPs have a significantly decreased antibacterial effect. However, an increase in the concentration of MNPs allows the attenuation of electrostatic repulsion through a bacterial surface saturation method (Abbaszadegan et al., 2015). After the adhesion onto the bacterial surface, MNPs can interact with the cells *via* two mechanisms, i.e, smaller MNPs penetrate directly into the cell, while the larger MNPs are retained outside the bacteria. In both situations, MNPs continuously release the metal ions, and these ions bind to the cell membrane structure that will destabilise the membrane potential and cause proton leakage. Cell wall destabilisation highly increases bacterial permeability and allows larger MNPs to enter the cell (Losasso et al., 2014).

There are a number of plant extracts and natural compounds isolated from plants that have antibacterial and antifungal activities. Most natural antimicrobial agents that target bacteria appear to disrupt membrane permeability, leading to membrane rupture and cell lysis. Furthermore, the inhibition of ergosterol biosynthesis, reduction of squalene epoxidase activity and fungal cell apoptosis are the specific mechanisms for antifungal, due to the structural differences between bacterial and fungal. However, not all mechanisms of action have been elucidated, and sometimes the mechanism may be indirect, stimulating the host immune system or inhibiting adhesion to the host cell.

**FIGURE 3:**
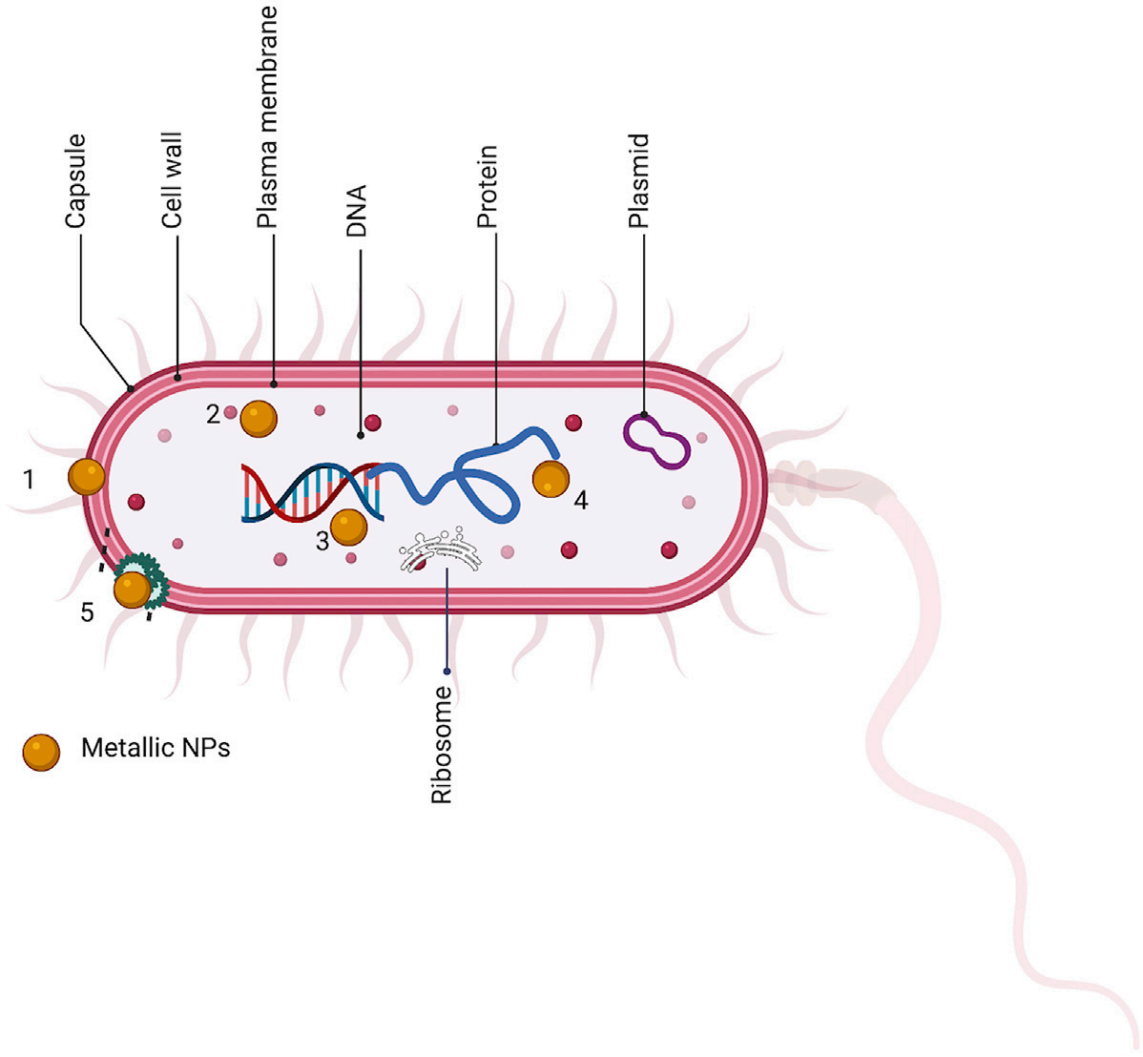
Sites of action of metal nanoparticle synthesized with natural product (MNps) on bacteria. Both components give synergically activity *via* similar mechanism of action bacterial cells. 1. bacterial efflux pump. 2. bacterial DNA. 3. bacterial ribosome. 4. bacterial protein. 5. bacterial cell walls integrity.

Therefore, due to the unique action of metal nanoparticles that working synergically with botanical drugs may be useful to overcome multi drug resistance bacteria. A proposed site of action MNPs in enhancing antimicrobial activity of natural products is shown in Figure 3.

Once inside the cell, MNPs and their metal ions interact with numerous structures and biomolecules such as proteins, lipids and DNA, resulting in cell dysfunction. MNPs are well known by their high capacity to produce ROS and free radicals such as hydrogen peroxide (H_2_O_2_), superoxide anion (O^−^), and hydroxyl radical (OH^•^). Even though ROS occur naturally in bacteria as a result of cellular respiration, under normal circumstances bacteria have defense mechanism such as glutathione (GSH), superoxide dismutase, and catalase that act as antioxidant enzymes and eliminate these toxic species. High concentrations of metal ions released by MNPs produce extreme levels of oxidative stress. However, antioxidant enzymes will remove some of the released metal ions and these are not enough to neutralise ROS and free radical by the MNPs amount (Qing et al., 2018). These species interact with respiratory chain proteins on the membrane and inactivate the enzyme due to their high affinity to phosphates, thiol and carboxyl groups (Gordon et al., 2010). Their linkage to phosphate groups of the enzyme will inhibit the phosphorylation of proteins which is important for enzymatic activation, and this process cause the inhibition of bacterial growth. Furthermore, dephosphorylation of tyrosine residues of protein was also been implicated in disruption of biosynthesis and transport of exopolysaccharide and capsular polysaccharide to the membrane that will cause the disruption of cell cycle (Dakal et al., 2016). In addition, metal ions can intercalate DNA strands forming complexes with nucleic acids between the purine and pyrimidine base pairs that cause the disruption of hydrogen between them (Qing et al., 2018) and cause the DNA damage.

### Characterisation MNPs

NPs’ physicochemical qualities influence their behaviour, biodistribution, safety, and efficacy. Thus, characterisation of metal NPs is critical for assessing the functional properties of the produced particles. Dynamic light scattering (DLS), zeta potential analyser, scanning electron microscopy (SEM), transmission electron microscopy (TEM), atomic force microscopy (AFM), X-ray diffractometry (XRD), Fourier transform infrared spectroscopy (FTIR) and UV-vis spectroscopy are some of the examples of technique used for NPs’ characterization. Combining these spectroscopic techniques offers information that could not be obtained with a single approach and helps in determine the quality of the synthesised nanoparticles.

The information such as size, size distribution, shape and surface chemistry are essential since these characteristics affect the absorption of NPs at the targeted site. Size of synthesised metal NPs is usually in the range of 5–100 nm and proven to enhance the antibacterial activity of plant extract loaded in NPs (Anbumani et al., 2022; Deivanathan and Prakash, 2022). The spectroscopy techniques such as DLS usually used to measure NPs size, size distribution and degree of aggregation.

Additionally, zeta potential analyser is used for determining the NPs surface charge. The surface charge above +30 mV and below −30 mV is generally regarded as targeted value to portrays lower degree of NPs aggregation and nanoparticle stability (Samimi et al., 2019). Shape is another NPs characteristic which can be measured using microscopy techniques such as SEM and TEM. TEM for instance, is a common technique to analyse NPs size, size distribution and shape. Other microscopy technique such as AFM can also being used to analyse NPs size, 3D-shape and elemental composition (Mourdikoudis et al., 2018) Usually after NPs synthesis, it will be characterised in terms of their crystal structure and lattice dimension using XRD. This technique was developed primarily to determine the 3D architectures and crystallinity of NPs.

FTIR spectroscopy is a method for determining NP structure and content. This method measures the absorption of electromagnetic radiation in the mid-infrared region (4,000–400 cm^−1^). FTIR analysis can be performed on both solid and liquid samples, and it has also been used to describe bacteria exposed to nanoparticles. The characteristics of functional groups and metabolites present on the surface of nanoparticles may be easily recognised using FTIR, which can occasionally aid in the reduction and stabilisation of nanoparticles (Nizamov et al., 2022).

**FIGURE 4:**
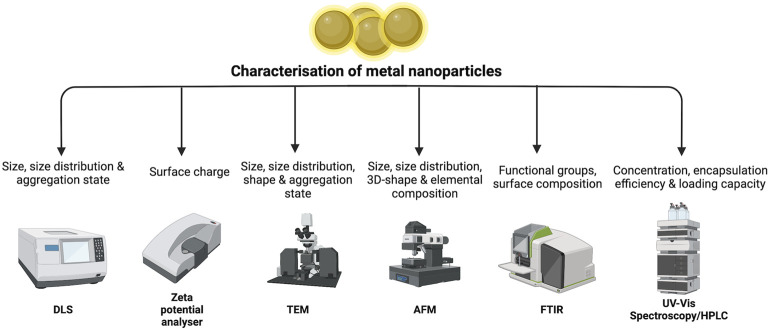
Summary of NPs characteristics and techniques.

UV-visible spectroscopy can be used to examine the scattering and absorption of light travelling through the material. This technique aids in the identification and characterisation of nanomaterials, as well as the determination of the stability of the nanoparticles generated. Other than that, HPLC provides more selective and specific measurement on the encapsulation efficiency, loading capacity and stability of plant extracts in NPs. Figure 4 summarizes the NPs characteristics and techniques used.

### Limitations and Prospects

The antimicrobial activity of plant extracts loaded in NPs is still unclear, like some are emphasized on bacterial oxidative stress, whereas other NPs such as MgO-NPs may not be associated with bacterial metabolism (Wang et al., 2017). The lack of evidence of mechanism of action of plant extracts itself become a major issue in this review. Another limitation is the complex structure of microorganisms not permitted to be stimulated from *in vitro* study to *in vivo* condition. The environmental issue especially to fish and plankton population should be considered another limitation of NPs, due the release of NPs to the environment. The NPs will be ingested by aquatic organisms and be accumulated in animals and further in the food chain (Maharramov et al., 2019).

The combination of two metal or bimetallic nanoparticles in formulation plant extract may increase the antimicrobial activity. The successful bimetallic nanoparticle of Ag-Fe (AgNPs and FeNPs) has been produced using redox reactions of aqueous Gardenia jasminoides. It showed a synergistic effect as bactericidal and fungicidal effects against Gram-positive, Gram-negative and yeast (Padilla-Cruz et al., 2021). On the other hand, the hybrid silver-iron nanoparticle containing aminolevulinic acid showed significant enhancement in their cytotoxic activity on MCF-7 cell lines (de Oliveira Gonçalves et al., 2020). In the case of multidrug resistance, NPs seems to be an alternative strategy to deliver antimicrobial agents to fight numerous antibiotic resistance bacteria.

## Conclusion

Metal nanoparticles alone have great potency as antimicrobial agents by showing different mechanisms of action. With the current development of drug delivery, the combination or green synthesis of natural products and in metal nanoparticles provide the advantage to produce synergistic activity of antimicrobial agents. Interestingly, there are not many purified compounds which possess antimicrobial agents that have been formulated with metal nanoparticles. It will give room to the researcher to do more research in this area. But this research would produce negative effects on the environment if not fully controlled. The use of uncontrolled metal nanoparticle can increase AMR incidents due to the effectiveness of metal nanoparticle in transformation of extracellular antibiotic resistant genes by 11-folds compared to the effects of antibiotics alone (Zhang et al., 2022).
